# Coping styles and mental health outcomes in partners who have experienced a perinatal loss: a longitudinal study

**DOI:** 10.3389/fpsyt.2026.1867729

**Published:** 2026-07-14

**Authors:** Luke Tarmey, Jane Iles, Lydia Poole

**Affiliations:** 1 School of Psychology, University of Surrey, Guildford, United Kingdom; 2 Department of Psychology, Royal Holloway, University of London, London, United Kingdom; 3 Centre for Psychiatry and Mental Health, Wolfson Institute of Population Health, Queen Mary University of London, London, United Kingdom

**Keywords:** perinatal loss, partners, grief, coping, depression, longitudinal, bereavement

## Abstract

Perinatal loss is common, but little is known about its impact on partners during the grieving process. This study examined psychological outcomes and coping strategies among recently bereaved partners (≤6 months post-loss; N = 73) at baseline (T1) and six-month follow-up (T2) via online survey. Participants were predominantly male (78%) and typically aged 25–44 (90%), and had experienced a range of perinatal losses (<20 weeks’ gestation to 28 days of life). Grief and depression symptoms were assessed using the Perinatal Grief Scale (PGS) and PHQ-9 respectively, and multivariate regression analyses examined the role of coping styles, demographic characteristics, and loss-related factors. Participants reported varying levels of grief and depression symptoms, with 38% displaying moderate-to-severe depression symptoms at follow-up. Across the cohort, participants reported using a range of coping strategies; however, avoidant coping was uniquely associated with higher grief and depression scores at baseline (PHQ-9: β = .30, p = .007; PGS: β = .49, p <.001). Avoidant coping also predicted poorer grief and depression outcomes at follow-up, although these relationships were no longer significant after controlling for baseline symptoms. Additionally, stillbirth, female gender, and younger age were associated with greater psychological distress at baseline across outcomes (β = .21–.32, p ≤.05). Associations between gender, stillbirth, and psychological outcomes remained at follow-up, although these did not reach conventional levels of statistical significance (β = .24–.40, p ≤.073). These findings highlight the psychological impact of perinatal loss on partners and underscore the importance of improving access to support for this group. Avoidant coping may represent a key target for intervention. Future research should further investigate factors influencing grief trajectories and identify effective forms of support for bereaved partners.

## 1 Introduction

Current data suggests as many as one in four pregnancies will result in perinatal loss, a term that encompasses losses occurring during pregnancy and shortly after birth, including miscarriage, early loss (<20 weeks gestation), still birth (>20 weeks), or neonatal loss (newborn through 28 days of life) (1). Globally, this equates to 2.6 million stillbirths and 2.7 million neonatal deaths per year (2), while miscarriage alone is estimated to occur in approximately 15% of recognised pregnancies (3). For many parents, the unexpected loss of a baby is a highly distressing event, and it is not uncommon for grief reactions to arise that include intense shock, numbness, sadness, denial, social withdrawal, and yearning for the baby (4, 5).

Whilst grief is considered a natural response to loss that often reduces in intensity over time, evidence suggests grief arising from perinatal loss may follow more complex trajectories, with symptoms persisting or intensifying for some individuals over several years. Across a two-year examination of perinatal grief, Lin and Lasker (6) revealed that less than half (41%) of the sample (N = 122) showed a decline in grief severity, with the remaining participants experiencing delayed resolution, worsening grief, or a persistently elevated grief response. Perinatal loss has also been associated with a range of mental health outcomes, predominantly studied among gestational mothers, including depression, anxiety, post-traumatic stress symptoms, and sleep difficulties (7–9). Increasing attention has therefore focused on identifying factors associated with differing psychological outcomes following perinatal loss.

For many, the centrality of parenthood as a core part of identity is thought to provide an additional layer of complexity to the grief process, as individuals must reconcile their anticipated role as parents with the reality of loss (10). Several factors have been associated with more severe grief responses following perinatal loss, including childlessness (6, 11), previous perinatal losses (12), poor quality social support (13, 14), older maternal age (15), and attachment difficulties (5, 16). There are mixed findings with respect to the impact of type of loss experienced on grief outcomes. Although greater emotional investment during pregnancy has been associated with increased grief severity (17), this relationship does not appear to be explained by gestational age alone, with studies reporting comparable grief and depression outcomes following miscarriage and stillbirth (11, 18, 19).

Perinatal loss has also been linked to greater incidence of complicated grief (CG), characterised by persistent and impairing grief reactions that interfere with adjustment following loss (20). Key features of CG include intense yearning, difficulty accepting the loss, and avoidance of loss reminders. These processes are thought to disrupt reintegration into life without the deceased and serve to maintain acute and protracted levels of distress (20). An avoidant grief response in the context of perinatal loss is likely compounded by wider cultural avoidance, as exemplified in a lack of clear cultural norms surrounding the death of a baby (21). Bereaved parents commonly report difficulty expressing the loss to others and creating mourning rituals (e.g., burial, funeral etc.) in societies characterised by silence around perinatal loss (22). This in turn may result in a sense of ‘disenfranchised grief’, where an invalidating social context contributes towards an isolative grief experience (23).

Whilst the majority of research in this area has focused on the maternal experience, increasing evidence suggests that partners can also experience significant psychological difficulties following perinatal loss (24). However, the way in which partners experience and respond to grief may differ. Societal expectations surrounding fathers’ role as a ‘strong supporter’ during pregnancy and following loss may contribute to difficulties expressing painful emotions and a greater reliance on more internalised or avoidant forms of coping, a pattern described as “double disenfranchised” grief (24). For example, men may engage in strategies such as increased workload or alcohol consumption as a means of managing their grief experience (25). Although less overt than emotional expressions of grief, avoidant coping responses may have important implications for psychological adjustment following loss. Previous research has highlighted avoidant coping as a predictor of complicated grief severity following traumatic loss (26), whereas approach-oriented strategies have been associated with greater post-traumatic growth and adaptation following loss (27).

Male partners may also experience additional barriers to accessing support, as services have historically focused on mother-centred care and forms of support that emphasise verbal emotional expression (28). This may partly reflect evidence suggesting that fathers report lower grief severity and fewer psychosocial difficulties compared with mothers following perinatal loss (29, 30). However, such differences may also reflect limitations in how grief is conceptualised and measured, with traditional grief measures emphasising emotionally overt expressions of loss (e.g., sadness, missing the baby, crying), while capturing fewer behavioural coping responses. As has been proposed, fathers may evidence a range of responses in the aftermath of a perinatal loss (24), including those which may be more ‘restoration-oriented’ (i.e., strategic avoidance, problem solving) as they attempt to find a way forward. Currently however, little is known about the prevalence of such strategies and how they might relate to grief and mental health outcomes over time.

Emerging research also suggests that non-gestational partners in same-sex couples may face additional complexities in their experience of perinatal loss. The journey towards parenthood may involve considerable emotional, financial, and practical investment, particularly where assisted reproductive technologies are involved (31). Furthermore, early opportunities to bond with the developing baby during fertility treatment may contribute towards a strong sense of parental identity before birth, as has been reported with IVF (32). Despite this, non-gestational partners may experience additional barriers to recognition and support, as their parental identity can be undermined through heteronormative assumptions and stigma (33). Accordingly, such individuals may experience added difficulty attaining positive social support from family and friends (34), alongside concerns about discrimination when approaching support services or healthcare professionals (35). Taken together, these findings underscore the need for a greater understanding of the impact of perinatal loss among non-gestational partners.

As part of the UK ‘NHS Long Term Plan’, Maternal Mental Health Services (MMHS) were launched in 2021 to improve access to psychological support following perinatal loss. Despite emerging evidence suggesting adverse outcomes among partners, access to specialist perinatal mental health support has historically focused on gestational mothers. As yet, there remains limited evidence regarding psychological outcomes among partners following perinatal loss, and greater understanding is needed to inform the development of inclusive support pathways. The present study therefore aims to investigate grief and depression outcomes among perinatally bereaved partners in the UK within the first year following loss, alongside the role of individual coping styles.

## 2 Materials and methods

### 2.1 Participants

An *a priori* power analysis was conducted using G*Power to determine the required sample size for multiple regression analysis. Assuming a medium effect size (f^2^ = 0.15), an alpha level of.05, and 10 predictors, the analysis indicated a minimum sample size of 118 participants to achieve adequate statistical power. As the final sample fell below this threshold, the analyses were interpreted as exploratory, with predictor selection limited to theoretically and empirically relevant variables to reduce model complexity.

Participants in the present study were 73 adults recruited within the first six months after perinatal bereavement, defined as loss occurring from conception to the first 28 days of life. Participants were recruited through social media advertising via third sector organisations, neonatal support groups, televised local news, and word of mouth. Participants were eligible if they identified as the partner of the person who carried the pregnancy, were fluent English speakers, and the loss occurred within the previous six months.

### 2.2 Patient and public involvement

A third-sector organisation specialising in perinatal loss support for couples provided consultation during the development of the study. Feedback informed the development of study materials, including recruitment materials, loss-related questionnaire items, and selection of outcome measures.

### 2.3 Procedure

Participants completed online questionnaires at recruitment (T1) and six months later (T2). Questionnaires were hosted via Qualtrics and accessed through a link included on study posters, emails, and social media posts. Participants were presented with an information sheet and consent form before completing the survey measures. During T1 data collection, participants provided a contact email address, which was used to send the T2 survey link. Participants were sent up to four reminder emails spaced approximately three weeks apart in the case of missing T2 data.

### 2.4 Measures

The T1 survey included demographic and loss-related items, a measure of coping style (Brief COPE; 36), a depression measure (PHQ-9; 37), and a grief symptom measure (Perinatal Grief Scale; PGS; 38). The T2 survey included the PHQ-9 and PGS only. Coping styles were measured at T1 to examine whether early coping responses following loss were associated with later psychological outcomes. A decision to omit the Brief COPE at T2 was made to reduce participant burden and maximise completion rates across both time points.

#### 2.4.1 Demographics and loss-related items

Participants were asked to record demographic information including their age, gender, ethnicity, occupational status, marital status, and highest level of education. For loss related items, participants selected the type of perinatal loss experienced, time elapsed since loss (1–6 months), number of previous perinatal losses, and any support received since loss (e.g., counselling, friends and family, support group etc.).

#### 2.4.2 PHQ-9

The PHQ-9 is a validated nine-item measure used to assess symptoms of depression (37). Total scores range from 0–27, with established cut-offs of 5, 10, 15, and 20 representing mild, moderate, moderately severe, and severe depression symptoms, respectively (37). The PHQ-9 has demonstrated strong psychometric properties across clinical populations, including individuals experiencing perinatal depression (39) and infertility-related difficulties (40).

#### 2.4.3 Grief severity measure: perinatal grief scale

The PGS is a validated 33-item measure used to assess grief symptoms following perinatal loss (38). Items are scored on a 5-point Likert scale ranging from 1 (strongly disagree) to 5 (strongly agree), with total scores ranging from 33–160. Higher scores indicate greater grief severity, with scores above 91 suggested to indicate significant vulnerability and greater likelihood of psychiatric morbidity (14).

The PGS comprises three 11-item subscales: active grief, difficulty coping, and despair (38). Subscale scores above 34, 30, and 27 respectively are considered elevated. The measure has demonstrated high internal consistency within perinatal loss populations, with reported Cronbach’s alpha values ranging from.92–.96 (41).

#### 2.4.4 Coping styles: brief COPE

The Brief-COPE (36) is a 28-item self-report questionnaire designed to measure different ways of coping with a stressful life event. The measure adopts a 4-point Likert scale (‘I haven’t been doing this at all’ to ‘I’ve been doing this a lot’) exploring a range of coping methods across 14 subscales comprised of two items each. As has previously been established across conceptual and empirical literature (26, 42), the subscales of the Brief-COPE can be further categorised to form three overarching coping styles. (1) Problem-focused coping describes strategies aimed at managing the stressful situation through instrumental action (e.g., planning, use of informational support). (2) Emotion-focused coping involves attempts to regulate emotional responses associated with the experience (e.g., venting, acceptance). (3) Avoidant coping refers to strategies aimed at reducing or avoiding unwanted thoughts and emotions associated with the distressing event (e.g., self-distraction, denial, use of substances).

To derive a score for each coping style, average scores were calculated for each respondent across the three overarching subscales, with higher scores indicating greater use of that coping style. The Brief-COPE has demonstrated good internal consistency when categorised into problem-focused, emotion-focused, and avoidant coping strategies, with Cronbach α ranging from.80 to.88 (26).

### 2.5 Analysis

Assessment of distributional assumptions indicated that skewness and kurtosis z-values for PHQ-9 and Problem-Focused Coping fell within acceptable limits (± 3.29; 30), supporting the assumption of normality. Emotion-Focused Coping exceeded this threshold (z = 4.60) and was treated as non-normally distributed in subsequent analyses.

Analyses were conducted in two stages: cross-sectional analyses examining relationships between study variables at baseline (T1), followed by longitudinal analyses examining changes in grief and depression outcomes at six-month follow-up (T2). Summary statistics were generated for study measures (PHQ-9, PGS, and Brief-COPE), followed by correlation analyses examining associations between grief, depression, and coping styles. Bivariate analyses were conducted to examine associations between demographic/loss-related factors and psychological outcomes.

Given the limited evidence regarding predictors of distress among partners following perinatal loss, an exploratory stepwise multiple regression approach was used at T1 to identify candidate predictors of depression (PHQ-9) and grief severity (PGS). This informed the selection of theoretically and empirically relevant variables for subsequent longitudinal analyses. The stepwise approach used both forward and backward elimination processes (F-to-enter p <.05, F-to-remove p >.051) to identify the most parsimonious predictive model (43).

Prospective predictors included gender, age, type of loss (stillbirth, miscarriage), previous perinatal loss (yes/no), time elapsed since loss (months), number of support sources accessed, and Brief-COPE scores (Avoidant, Problem-Focused, Emotion-Focused). To account for the exploratory nature of analyses and sample size considerations, the number of predictors included within longitudinal models was restricted. Age was dichotomised (<35, >35) due to small numbers within some age categories. Tolerance and variance inflation factor values indicated multicollinearity was not a concern.

In the longitudinal analysis, paired-sample t-tests were used to assess differences in depression (PHQ-9) and grief severity (PGS) scores between T1 and T2. Frequencies of change characteristics were generated to describe individual symptom trajectories, categorised as decreasing, increasing, or unchanged severity. Given differences in scale ranges, meaningful change thresholds were defined separately for each measure. For the PHQ-9 (range: 0–27), a change of >1 point in either direction was used to indicate change in symptom severity. For the PGS (range: 33–160), a threshold of >5 points was selected due to the larger range of possible scores. Scores not exceeding these thresholds were categorised as unchanged.

Finally, hierarchical multiple regression analyses were performed to examine whether early avoidant coping responses (Brief-COPE) at T1 predicted depression (PHQ-9) and grief severity (PGS) scores at T2. Avoidant coping was selected as the primary independent variable as it emerged uniquely among coping styles as associated with grief and depression severity at T1, and was theoretically relevant based on previous research (20, 26).

Covariates were selected based on findings from cross-sectional analyses and entered sequentially to examine their contribution to outcomes. Avoidant coping was entered at Step 1, followed by relevant demographic and loss-related factors at Step 2, including time elapsed since loss (months), stillbirth (1 = yes, 2 = no), age (<35, >35), and gender (1 = male, 2 = female). Baseline symptom scores (PHQ-9 T1 or PGS T1) were entered at Step 3 to examine whether avoidant coping predicted outcomes after accounting for initial symptom severity.

## 3 Results

### 3.1 Response rates

Seventy-three participants completed baseline measures (T1), with minimal missing data on the PHQ-9 (n = 1) and Brief-COPE (n = 2). Of these, 52 participants (71%) completed follow-up measures (T2) and were included in longitudinal analyses.

### 3.2 Sample characteristics

Participant demographic and loss-related characteristics for the total sample (T1) and participants retained at follow-up (T2) are displayed in **Tables 1**, **2**. Participants completed baseline measures an average of 4.11 months post-loss (SD = 2.01, range = 1–6 months), with follow-up completed an average of 9.88 months following loss (SD = 1.84, range = 7–13 months).

**Table 1:** Frequencies of loss related factors.

	Cross sectional sample		Longitudinal sample	
Characteristic	N (73)	%	N (52)	%
Type of loss				
Miscarriage	26	35.6	17	32.7
Ectopic Pregnancy	3	4.1	1	1.92
Still Birth	23	31.5	16	30.77
Neonatal Death	8	10.9	7	13.46
TFMR	13	17.8	11	21.15
Support received				
Counselling	30	41.1	20	38.46
Friends and Family	48	65.8	37	71.15
Support Group	14	19.2	9	17.31
Charity	20	27.4	15	28.85
Bereavement Midwife	3	4.2	1	1.92

T1				
	Mean	Median	Mean	Median
	(SD)	(Range)	(SD)	(Range)
Time since loss (months)	4.11 (2.01)	5 (1-6)	3.9 (2.08)	4 (1-6)
Number sources of support received	1.60 (1.19)	1.0 (0-4)	1.63 (1.19)	2.0 (0-4)
Previous losses	0.56 (1.04)	0 (0-5)	0.46 (0.90)	(0) (0-5)

TFMR. Termination for Medical Reasons.

**Table 2:** Frequencies of sample demographics.

	Cross sectional sample		Longitudinal sample	
Characteristic	N (73)	%	N (52)	%
Age bracket				
<18	1	1.4	1	1.90
18-24	2	2.7	1	2.70
25-34	32	43.8	20	38.50
35-44	34	46.6	27	51.90
45-54	4	5.5	3	5.80
Gender				
Male	57	78.1	38	73.10
Female	15	20.5	14	26.90
Non-Binary	1	1.4		
Ethnicity				
White	66	90.4	48	92.30
Black	1	1.4	1	1.90
Asian	3	4.1	2	3.80
Arab	1	1.4	1	1.90
Mixed	2	2.9		
Occupational status				
Employed	66	90.4	47	90.38
Unemployed	4	5.5	3	5.77
Retired	1	1.4	0	
Other	2	2.7	2	3.84
Marital status				
Married	49	67.1	35	67.30
Co-habiting	19	26.0	12	23.08
Widowed	1	1.4	0	
Divorced/Separated	2	2.7	2	3.84
Never been married	2	2.7	2	3.84
Highest level of education				
Primary	1	1.4	0	
Some Secondary	2	2.7	2	3.90
Completed Secondary	7	9.6	6	11.54
Vocational	15	20.5	10	19.60
Some University	9	12.3	7	13.73
Bachelors Degree	23	31.5	17	33.30
Graduate Degree	14	19.2	8	15.69

Comparisons between participants retained at T2 and those who completed T1 only indicated no significant differences across study variables (p >.05).

### 3.3 Cross sectional analysis

#### 3.3.1 Outcome variables at T1 – levels of depression, grief severity and coping styles among partners within six months post loss

A full breakdown of summary statistics across outcome variables at T1 is presented in **Table 3**. The mean PHQ-9 severity score at T1 was 10.96 (SD = 6.67), falling within the moderate depression range. Overall, 57.5% (n = 42) of participants scored within the mild to severe depression range (see **Table 4**).

**Table 3:** Descriptives of PHQ-9, PGS and Brief-COPE scores at T1.

Outcome	Clinical significance criteria	N	Mean	SD	IQR	Min-max (range)	α
PHQ-9	Scores of 5, 10, 15, and 20 represent mild, moderate, moderately severe, and severe depression, respectively.	72	10.96	6.67	8	0-27 (27)	0.89
Total PGS	> 91 = severe	73	103.95	24.01	31	48-155 (107)	0.95
PGS: Active Grief	> 34 = severe	73	39.97	7.97	9	14-54 (40)	0.85
PGS: Difficulty Coping	> 30 = severe	73	34.74	8.50	11	18-53 (35)	0.90
PGS: Despair	> 27 = severe	73	29.23	9.41	14	14-50 (36)	0.89
Emotion Focused Coping	N/A	71	25.72	5.64	6	17-45 (28)	0.72
Problem Focused Coping	N/A	71	18.83	5.64	9	10-32 (22)	0.85
Avoidant Coping	N/A	71	15.78	4.07	5	8-31 (23)	0.73

**Table 4:** Categorical severity frequencies for PHQ-9 at T1 and T2.

	T1 (N = 72		T2 (N = 52)	
Clinical Significance	N	%	N	%
None (0-4)	13	17.8	18	34.6
Mild (5-9)	17	23.3	18	34.6
Moderate (10-14)	26	35.6	8	15.4
Moderately Severe (15-19)	7	9.6	5	9.6
Severe (20-27)	9	12.3	3	5.8

The mean total PGS score was 103.95 (SD = 24.01), exceeding the threshold associated with increased vulnerability and likelihood of psychiatric morbidity. Mean scores across all PGS subscales fell within the high range (see **Table 3**).

On the Brief-COPE, the coping style most strongly adhered to was Emotion Focused (M = 25.72, SD = 5.64, Range = 17-45), followed by Problem Focused (M = 18.83, SD = 5.64, Range = 10-32), and Avoidant (M = 15.79, SD = 4.07, Range = 8-31).

#### 3.3.2 Relationships between variables at T1

As shown in **Table 5**, there was a strong positive correlation between total grief severity (PGS-Total) and depression levels (PHQ-9-Total) [*r* = .80, p <.001]. PHQ-9 Total was also strongly correlated with each of the PGS Grief Subscales, Active Grief [*r* = .74, p <.001], Difficulty Coping [*r* = .80, p <.001], and Despair [*r* = .69, p <.001]. Due to high interscale correlations between PGS subscales (r = .74–.79, p <.001), the Total PGS score was used as the primary measure of grief severity in subsequent analyses.

**Table 5:** Pearson’s correlation matrix showing associations between variables at T1.

Variable	1	2	3	4	5	6	7 ^	8
1. PHQ-9-Total		0.74**	0.80**	0.69**	0.80**	-0.02	0.24	0.36**
2. PGS-Active			0.80**	0.79**	0.93**	-0.14	0.14	0.44**
3. PGS-Difficulty Coping				0.78**	0.93**	-0.08	0.18	0.44**
4. PGS-Despair [0-55]					0.93**	-0.03	0.28**	0.56**
5. PGS-Total						-0.09	0.22	0.52**
6. Brief-COPE-Problem							0.56**	0.12
7. Brief-COPE-Emotion^								0.34**
8.Brief-COPE-Avoidant								

**p < 0.01, ^Spearman's Rho.

For coping styles assessed using the Brief-COPE, only Avoidant Coping was associated with depression scores on PHQ-9 [*r* = .36, p <.05], whilst also being positively correlated with Total Grief [*r* = .52, p <.001]. Moderate strength positive intercorrelations were observed within subscales of the Brief COPE [p <.001], with the exception of Problem Focused Coping, which was positively associated only with Emotion Focused Coping [*r* = .56, p <.001].

#### 3.3.3 Bivariate analyses exploring demographic and loss related characteristics on outcome variables at T1

The bivariate analyses revealed several significant associations between demographic and loss-related factors and outcome variables (PGS, PHQ-9, Brief COPE) at T1. Female partners reported higher total mean grief scores [M = 116.14, SD = 25.67] compared to male partners [M = 100.68, SD = 22.93; t(70) = -2.27, p <.05]. Time elapsed since loss was negatively correlated with PHQ-9 scores, with more recent losses associated with higher depression scores [ρ = -0.32, p <.05]. Age was negatively correlated with PHQ-9 scores [ρ = -.28, p <.05], with younger participants reporting higher depression severity. Participants without a previous perinatal loss reported higher levels of avoidant coping [M = 16.5, SD = 4.34] than those with previous loss experience [M = 14.05, SD = 2.71; t(69) = 2.41, p <.05]. Number of previous losses was negatively correlated with avoidant coping [ρ = -0.31, p <.05], while number of support sources accessed was positively associated with Problem Focused Coping [ρ = .30, p <.05].

#### 3.3.4 Cross-sectional multivariate analysis

(a) Modelling Depression Severity (PHQ-9) at T1.

Starting with 10 candidate variables (age (>35, <35), gender (male, female), stillbirth (yes, no), miscarriage (yes, no), experience of previous loss (yes/no), time elapsed since loss (months), number of support sources accessed, and the three Brief-COPE scales), a final regression model was derived containing four significant predictors of PHQ-9 scores (F(4,64) = 7.17, p <.001). The adjusted R^2^ value was.266, with the model explaining 26.6% of variance in PHQ-9 scores.

As shown in **Table 6**, significant predictors were gender (B = 3.38, p = .049), stillbirth experience (B = -3.20, p = .013), age (B = -4.24, p = .005), and Avoidant Coping scores (B = 0.49, p = .006). No first-order interaction effects were observed.

**Table 6:** Stepwise multiple regression analysis predicting total PHQ-9 scores at T1: final model.

	B	SE B	95% CI	β	p-value
Constant	7.86	4.70	[-1.51, 17.28]		
Gender	3.38	1.69	[0.002, 6.75]	0.21	0.049
Stillbirth	-3.20	1.54	[-6.99, - 0.84]	-0.28	0.013
Age 35+	-4.24	1.46	[-7.14, -1.33]	-0.32	0.005
Brief-COPE Avoidance T1	0.49	0.18	[0.14, 0.84]	0.30	0.007

Gender (1 = male, 2 = female), Stillbirth (1 = yes, 2 = no), Age (> 35 = 1, < 35 = 2).

Adjusted *R^2^* = .23, *p <* 0.001.

(b) Modelling Total Grief Severity (PGS) at T1.

From the same pool of 10 candidate variables, a total of 3 variables were upheld as significant predictors contributing to Total PGS scores (F (3, 66) = 13.45, p <.001). The adjusted R^2^ value was.35, with the model accounting for 35.1% of variance in total PGS score at T1. Significant predictors included gender (B = 13.44, p = .021), stillbirth experience (B = -11.67, p = .022), and Brief-COPE Avoidance scores (B = 2.94, p <.001). No first-order interaction effects were obtained (see **Table 7** for model summary).

**Table 7:** Stepwise multiple regression analysis predicting total PGS scores at T1: final model.

	B	SE B	95% CI	β	p-value
Constant	60.88	14.53	[31.87, 89.89]		
Gender	13.44	5.70	[2.05, 24.83]	0.23	0.021
Stillbirth	-11.67	4.99	[-21.63, -1.72]	-0.23	0.022
Brief-COPE Avoidance T1	2.94	0.59	[1.78, 4.11]	0.49	<.001

Gender (1 = male, 2 = female), Stillbirth (1, = yes, 2 = no). Adjusted *R^2^* = .351, *p <* 0.001.

Longitudinal analysis.

##### 3.3.4.1 Changes (T2-T1) in outcomes scores (PHQ-9, PGS)

Mean scores and the direction of changes in scores over time can be found in **Table 8**. Mean PHQ-9 depression scores significantly decreased over time (p <.001), along with total grief severity (PGS; p <.05). At T2, the mean depression score (7.77, SD = 5.70) fell within the mild range (5–9), although 30.77% of participants (n = 16) continued to score within the moderate to severe range (see **Table 4**). Although grief intensity reduced over time, the mean total severity score at T2 (95.65, SD = 25.37) remained above the clinical threshold (>91).

**Table 8:** Change in outcome scores between T1 - T2.

	T1			T2				
Outcome	N	Mean	SD	Mean	SD	Mean change Score	SD change	Cohen's d
PHQ-9	52	10.65	6.67	7.77	5.70	-2.88**	4.33	0.67
Total PGS	51	102.18	23.06	95.65	25.37	-6.54*	15.19	0.14
PGS: Active Grief	51	39.98	7.35	37.00	7.99	-2.98**	5.36	0.56
PGS: Difficulty Coping	51	33.88	8.31	31.98	9.43	-1.90*	5.92	0.32
PGS: Despair	51	28.30	9.13	26.67	9.83	-1.64*	7.14	0.23

*p < 0.05, **p < 0.001, Paired samples *t*-test.

Frequencies of change characteristics are displayed in **Table 9** for PHQ-9 and Total Grief. The majority of participants evidenced a reduction in severity scores over time for both PHQ-9 (69.2%, n = 36) and PGS (51%, n = 26), although a proportion of participants showed unchanged or increased symptom severity (see **Table 9**).

**Table 9:** Frequencies of change characteristics for depressive symptoms and grief.

	Total PHQ-9*		Total PGS**	
Severity change	N	%	N	%
Increasing	5	9.6	11	21.6
Unchanged	11	21.2	14	27.5
Decreasing	36	69.2	26	51
Total	52	100.0	51	100

*/- 1, */- 5.

##### 3.3.4.2 Multivariate analyses of grief outcomes over time

(a) Modelling Depression Severity at Follow-Up (PHQ-9 T2 Score).

In step one, Brief-COPE Avoidance at T1 was entered alone as the predictor. The analysis showed that this factor significantly predicted PHQ-9 total scores at T2 (B = 0.50, p = .034), accounting for 7.1% of the variance in scores (Adjusted R^2^ = .071), F(1, 48) = 4.77, p = .034. In the second step, co-variates (age, gender, stillbirth, and time elapsed since loss) were entered. This model explained an additional 20.1% of the variance (Adjusted R^2^ = .272), F(5, 44) = 4.66, p = .002. The significant predictors in this model were gender (B = 4.56, p = .005) and Brief-COPE Avoidance at T1 (B = 0.42, p = .049). In the third step, the PHQ-9 total score at T1 was added to the model to control for participants’ baseline severity. This model explained an additional 30.4% of the variance (Adjusted R^2^ = .576), F(6, 43) = 12.08, p <.001. The only significant predictor in this final model was PHQ-9 total severity score at T1 (B = .61, p <.001) (see **Table 10** for summary).

**Table 10:** Hierarchical multiple regression analysis predicting T2 PHQ-9 scores.

Model	*B*	SE	95 % CI	*β*	*p* value
Step 1					
Brief-COPE Avoidance T1	0.50	0.23	[.04,.96]	0.30	0.034
Step 2					
Brief-COPE Avoidance T1	0.42	0.21	[.003,.83]	0.25	0.049
Age	-2.01	1.52	[-5.07, 1.06]	-0.18	0.194
Gender	4.56	1.55	[1.44, 7.67]	0.36	0.005
Stillbirth	-2.91	1.59	[-6.10,.28]	-0.24	0.073
Time elapsed since loss	-0.52	0.34	[-1.21,.17]	-0.19	0.136
Step 3					
Brief-COPE Avoidance T1	0.16	0.16	[-.16, 0.49]	0.10	0.321
Age	0.16	1.22	[-2.31, 2.62]	0.01	0.897
Gender	2.07	1.26	[-0.47, 4.61]	0.17	0.107
Stillbirth	-1.59	1.23	[-4.07, 0.90]	-0.13	0.205
Time elapsed since loss	0.12	0.29	[-0.45, 0.70]	0.04	0.671
PHQ-9 T1	0.61	0.11	[0.39, 0.82]	0.68	<0.001

Age (> 35 = 1, < 35 = 2), Gender (1 = male, 2 = female), Stillbirth (1, = yes, 2 = no).

(b) Modelling Total Grief Severity at Follow-Up (PGS T2 Score).

In the first step, Brief-COPE Avoidance score at T1 was entered alone as the predictor. The results indicated that this variable significantly predicted PGS T2 score (B = 2.74, p = .008), explaining 12.1% of the variance in grief scores (Adjusted R^2^ = .121), F(1, 47) = 7.64, p = .008. In the second step, additional co-variates were entered, including time elapsed since loss (months), gender (male, female), stillbirth (yes, no), and age (<35, >35). The second model explained an additional 20.8% of the variance (Adjusted R^2^ = .329), F(5, 43) = 5.70, p <.001. Significant predictors in this model were gender (B = 22.36, p = .002), stillbirth (B = -15.43, p = .028), and Brief-COPE Avoidance at T1 (B = 2.38, p = .010). In the final step, participants’ PGS total score at T1 was added into the model to control for baseline grief severity. This model explained an additional 36.1% of the variance (Adjusted R^2^ = .690), F(6, 42) = 18.85, p <.001. The only significant predictor in the final model was total grief score at T1 (B = .83, p <.001) (see **Table 11** for summary).

**Table 11:** Hierarchical multiple regression analysis predicting T2 PGS scores.

Model	*B*	SE	*β*	95 % CI	*p* value
Step 1					
Brief-COPE Avoidance T1	2.74	0.99	0.37	[.75, 4.74]	0.008
Step 2					
Brief-COPE Avoidance T1	2.38	0.88	0.33	[.61, 4.16]	0.010
Age	-6.72	6.64	-0.13	[-20.12, 6.67]	0.317
Gender	22.36	6.65	0.40	[8.94, 35.78]	0.002
Stillbirth	-15.43	6.80	-0.29	[-29.15, -1.72]	0.028
Time elapsed since loss	-0.79	1.50	-0.06	[-3.81, 2.22]	0.599
Step 3					
Brief-COPE Avoidance T1	-0.08	0.70	-0.01	[-1.47, 1.31]	0.911
Age	-1.94	4.56	-0.04	[-11.14, 7.26]	0.673
Gender	7.61	4.97	0.14	[-2.41, 17.63]	0.133
Stillbirth	-8.05	4.73	-0.15	[-17.60, 1.50]	0.096
Time elapsed since loss	-0.05	1.02	-0.004	[-2.11, 2.01]	0.962
PGS T1 score	0.83	0.12	0.75	[.60, 1.06]	<.001

Age (> 35 = 1, < 35 = 2), Gender (1 = male, 2 = female), Stillbirth (1, = yes, 2 = no). Time elapsed since loss (months). Step 1 adjusted R^2^ = .121, p = 0.008. Step 2 adjusted R^2^ = .329, p <.001. Step 3 adjusted R^2^ = .690, p <.001.

## 4 Discussion

This study sought to address two main research aims. Firstly, it aimed to establish the psychological impact of perinatal loss on partners over a six-month period, assessed through measures of grief severity (PGS; 38) and depression (PHQ-9; 37). Consistent with initial predictions, partners displayed considerable variation in their responses following loss. Whilst grief and depression symptoms generally reduced over time, this was not a universal pattern, with a proportion of partners continuing to experience clinically significant distress at follow-up. These findings contribute to growing evidence that, like gestational parents, partners can experience significant and enduring psychological consequences following perinatal loss.

Partners also evidenced a range of early coping responses following perinatal loss. Despite previous literature highlighting avoidant coping as a response commonly associated with bereaved fathers (24), findings from the present study suggest that partners utilise a broader range of coping responses following loss. This variation supports previous research cautioning against typifying partners’ grief experiences into a single mode of coping (24), highlighting instead the complex and varied nature of individual responses. These findings may also align with the Dual Process Model of Coping with Bereavement (44), whereby individuals oscillate between loss-oriented and restoration-oriented coping strategies during adjustment to loss, rather than remain fixed in any one coping style as theorised by traditional ‘stage models’ of grief (45).

The second research aim sought to clarify the relationships between variables to understand what factors are associated with differing grief trajectories among partners. Initial exploration revealed that most coping styles were largely independent of grief and depression scores. This distinction may support the exploration of coping alongside traditional symptom-based measures to capture a broader range of responses to perinatal loss. For instance, strong adherence to problem solving strategies in the wake of perinatal loss may represent meaningful attempts to engage and adapt to grief for some partners, highlighting that meaningful responses to loss may extend beyond overt emotional expressions of distress.

An exception to this pattern was avoidant coping, which emerged as the coping strategy most consistently associated with poorer psychological outcomes, showing associations with greater grief and depression severity both cross-sectionally and at six-month follow-up. This finding aligns with literature highlighting experiential avoidance as a process involved in the maintenance of distress following bereavement. Avoidance may contribute to poorer adjustment through multiple pathways, including increased social isolation and withdrawal (46), increased emotional salience of avoided loss-related content (47), and ruminative processes that interfere with emotional processing of the loss (48). However, the direction of this relationship remains unclear; avoidant coping may contribute towards ongoing distress, but may also plausibly emerge as a response to experiences of loss perceived as more distressing and thus harder to approach.

Additional loss-related and demographic factors were also associated with outcomes, including stillbirth, younger age, and female gender. The association between stillbirth and greater grief severity may reflect the particularly distressing nature of later pregnancy losses, where greater time to develop attachment to the baby and prepare for parenthood may intensify the experience of loss. Similarly, while younger age was associated with greater grief and depression severity among partners, further research is needed to understand whether factors associated with age operate differently for partners compared with gestational parents. One possibility is that older partners may have developed greater resources for managing difficult life events (49), while younger partners may experience the sudden and unexpected nature of perinatal loss as particularly disruptive to their expectations surrounding parenthood (50). However, findings should be interpreted cautiously given the exploratory nature of analyses and limited sample size.

Despite partial support for the influence of individual factors in predicting symptom severity at follow-up, partners’ baseline grief and depression scores emerged as the strongest predictors of longer-term outcomes. This may reflect temporal dynamics often observed in longitudinal studies investigating responses to traumatic events, whereby initial symptom severity accounts for a substantial proportion of later outcomes (51). Nevertheless, the finding that early symptom severity was closely associated with later distress may have important clinical implications, highlighting the potential value of identifying and supporting partners experiencing high levels of distress in the initial aftermath of loss.

However, as not all individuals in the sample followed a normative (i.e., decreasing) pattern, the present findings may also highlight a limitation of multiple linear regression in detecting factors associated with heterogeneous grief responses. This approach assumes a single average trajectory for all individuals while also assuming that past values of the dependent variable (e.g., T1 grief) uniformly affect future values (e.g., T2 score) across all individuals. It may be the case however that some factors (e.g., coping style) are associated with certain grief trajectories among partners but not others. One alternative approach to explore this is Latent Growth Mixture Modelling (LGMM), as used by Smith and Ehlers (52) in their exploration of cognitive predictors of grief. This approach allows for distinct subgroups, or ‘latent classes’, of individuals to be identified relative to a range of estimated parameters, including initial levels of grief severity and rate of change over time. Predictor variables can then be modelled against the likelihood of class membership (e.g., low unchanged grief, fast increasing). Researchers wishing to further explore the factors associated with differing trajectories of bereaved partners may wish to adopt such an approach on a larger scale.

A key limitation of the present study was the sample size, particularly within longitudinal analyses, which may have reduced statistical power to detect significant associations. Recruitment challenges should be considered within the context of partners following perinatal loss being described as a ‘hard-to-reach’ population (53). This study drew primarily on third-sector support groups to recruit participants; however, due to a lack of societal recognition for their status as grievers, partners may be less likely to approach such services in the aftermath of loss, which often tend to focus on supporting gestational ‘mothers’ (28). Additionally, sampling predominantly through targeted baby loss groups may have contributed towards a demographically homogenous sample, as most participants were White, well-educated, and employed. As a result, the data collected here may reflect only a specific subsection of the wider population of partners experiencing perinatal loss. Future research may benefit from broader recruitment strategies, including earlier opportunities within the perinatal loss pathway (e.g., maternity settings), to engage partners from a wider range of backgrounds and loss experiences.

An additional limitation of the present study is that coping styles were only measured at T1. Whilst this decision was made to reduce participant burden and maximise follow-up completion, it limits understanding of whether coping represents a relatively stable individual tendency that may pre-date the loss, or a contextually meaningful response following perinatal loss. Future research assessing coping styles across multiple timepoints may clarify whether coping responses change during the grieving process and whether specific strategies serve different functions depending on the context in which they occur (e.g., Dual Process Model; 44). Given the role of avoidance processes in trauma-related symptoms, future studies may also benefit from examining post-traumatic stress responses following perinatal loss.

### 4.1 Clinical implications

Despite these limitations, the present study contributes some important findings with clinical implications. Firstly, it adds to a growing evidence base that like gestational parents, partners also experience severe grief and depressive reactions that remain clinically significant beyond the initial six months post perinatal loss. This is important since many existing public support services are currently not commissioned to work with partners, who have been widely regarded as demonstrating less severe grief responses. Furthermore, the association between avoidant coping and grief and depression severity, services should not assume that individuals who may present as reluctant to seek support are less affected by the loss; rather, alternative and more acceptable forms of support may be required. Services should therefore adopt flexible and inclusive approaches that recognise the heterogeneity of partners’ responses to loss and facilitate access to a range of support options, including both formal interventions and community-based or peer-led initiatives. Additionally, strengthening collaboration between statutory services and third-sector organisations may help to improve access and engagement, through increased awareness among healthcare professionals and smoother referral pathways.

Greater awareness may also be required to support female partners, who may be at increased risk of distress and may encounter additional barriers within healthcare systems. Providing inclusive, trauma-informed care, such as the use of affirming language (54), culturally competent practice (55), and recognition of partners’ parental status (56), may help to reduce the risk of retraumatisation and support engagement with services. More broadly, across partners, avoidant coping may represent a key target for intervention; however, addressing this is unlikely to be solely an individual responsibility given that partners’ experiences of loss are currently situated within a broader avoidant social context (24). Rather, a coordinated approach across clinical, community, and policy levels is likely required to foster environments that support openness and engagement with grief. This may include addressing systemic inequalities in partners’ rights during the loss experience, such as access to bereavement leave, and moving away from treating parents in isolation according to traditional gender roles. Recognising partners as legitimate participants throughout the loss experience may help to create a less isolating social context that is more conducive to the wellbeing of both parents, irrespective of who carried the baby.

### 4.2 Conclusion

This study has built upon previous research attesting to the impact of perinatal loss on partners, revealing that like gestational parents, partners can also be deeply affected in the aftermath of losing a baby. Evidence supports considerable variation in responses however, evident through a wide range of scores and the use of multiple coping strategies. Avoidant coping appears to be a strategy predictive of more severe distress responses. Furthermore, there is some initial support for an association between female partners and increased vulnerability to perinatal loss, whilst stillbirth and younger age also appear to be factors associated with more severe outcomes. Further research is warranted to explore the impact of these variables, along with coping propensity over time. Finally, clinicians and policy makers are advised to show greater awareness and inclusivity towards partners, who may likely benefit from additional support to manage their grief.

## Data Availability

The raw data supporting the conclusions of this article will be made available by the authors, without undue reservation.
